# Efficient and Scalable Graph Similarity Joins in MapReduce

**DOI:** 10.1155/2014/749028

**Published:** 2014-07-08

**Authors:** Yifan Chen, Xiang Zhao, Chuan Xiao, Weiming Zhang, Jiuyang Tang

**Affiliations:** ^1^College of Information System and Management, National University of Defense Technology, Changsha 410073, China; ^2^Nagoya University, Nagoya, Japan

## Abstract

Along with the emergence of massive graph-modeled data, it is of great importance to investigate graph similarity joins due to their wide applications for multiple purposes, including data cleaning, and near duplicate detection. This paper considers graph similarity joins with edit distance constraints, which return pairs of graphs such that their edit distances are no larger than a given threshold. Leveraging the MapReduce programming model, we propose MGSJoin, a scalable algorithm following the filtering-verification framework for efficient graph similarity joins. It relies on counting overlapping graph signatures for filtering out nonpromising candidates. With the potential issue of too many key-value pairs in the filtering phase, spectral Bloom filters are introduced to reduce the number of key-value pairs. Furthermore, we integrate the multiway join strategy to boost the verification, where a MapReduce-based method is proposed for GED calculation. The superior efficiency and scalability of the proposed algorithms are demonstrated by extensive experimental results.

## 1. Introduction

As the most commonly used abstract data structure, graphs have been widely used for modeling the data in the fields of bioinformatics, multimedia, social networking, and the like. As a consequence, efforts were dedicated to various problems in managing and analyzing graph data, for example, frequent subgraph mining [1], structure search and indexing [2, 3], similarity search [4, 5], and so forth.

This paper focuses on graph similarity join, one basic operation for processing graph data. Given two graph object sets *R* and *S* and a distance threshold, a graph similarity join returns all the pairs of graph objects, respectively, from *R* and *S*, the distances of which are no larger than the threshold. Graph similarity join has a wide spectral of applications, especially in preprocessing of graph mining, for example, structural data cleaning and near replicate structure detection.

The most widely applied measure for determining graph similarity is graph edit distance (GED) [6, 7]. Compared with alternative measures, GED has at least three advantages: (1) it allows changes in both vertices and edges; (2) it reflects the topological information of graphs; (3) it is a metric that can be applied to any type of graphs. Consequently, we employ GED to quantify graph similarity in this paper. It is shown that exact computation of GED is NP-hard [8].

The state-of-the-art algorithm for graph similarity join is GSimJoin [9], which adopts the filtering-verification framework. In particular, signatures are generated for every graph with path-based *q*-gram approach for count filtering (cf.  Section 2.2). In the phase of verification, GED computation is invoked for candidate pairs via an A*-based algorithm. GSimJoin is an in-memory algorithm, the performance and scalability of which are restricted to the available memory of a machine. The dataset size presented in the experimental study is limited in the thousands, since a graph similarity join operation in the worst case needs *O*(|*R*||*S*|) count filtering condition checks and similarity computations thereafter. The era of big data calls for scalable algorithms to support large-scale data processing. This paper attempts to address the challenges on massive graph data.

MapReduce is a well-known programming framework to facilitate processing large-scale data in parallel [10]. MassJoin [11] is a MapReduce-based algorithm for similarity join on strings. Nonetheless, there has been no existing distributed algorithm for graph similarity joins. Inspired by [11], this paper investigates graph similarity joins based on MapReduce.

We firstly propose MGSJoin, a MapReduce-based algorithm following the filtering-verification framework. It employs signatures of path-base *q*-grams as keys and the corresponding graphs as values, forming* key-value pairs*. Through filtering, the graph pairs, whose common signatures are less than the threshold given by count filtering condition, are filtered out. The remaining pairs constitute the candidate set, sent to verification thereafter. Due to the potentially large number of key-value pairs that may incur large communication cost, we incorporate the* Bloom filter* technique by adding generated signatures to spectral Bloom filters. This effectively reduces the number of intermediate key-value pairs and, thus, the complexity of network transmission, while the filtering capacity is mostly preserved. Furthermore, we employ* multiway join* to improve the verification phase by condensing two MapReduce rounds into one while devising a MapReduce-based method to calculate GED.

To the best of our knowledge, it is among the first attempts to present a MapReduce-based graph similarity join algorithm. Our contribution can be summarized as follows.

(i)  We redesign the current in-memory graph similarity join algorithm and adapt it to the MapReduce framework. The resulting baseline algorithm is capable of processing large-scale graph datasets.
(ii)  We propose to use Bloom filters to reduce intermediate key-value pairs in the filtering phase while sacrificing little filtering capacity. Besides, we present a multiway join optimized verification strategy such that the number of required MapReduce rounds is reduced too. Moreover, a MapReduce-based method is designed for GED calculation, which can handle the calculation for large graphs.
(iii)  We implement the proposed algorithm MGSJoin and conduct a wide range of experiments on a real dataset. The results show that both the efficiency and the scalability of MGSJoin are superior to the current solutions.

This paper is constructed as follows. In Section 2, problem definition and background are provided. We propose the basic algorithm in Section 3, integrate Bloom filters in Section 4, and optimize the verification in Section 5.  Section 6 lists the experimental results and analyses. Related works are described in Section 7, followed by conclusion in Section 8.

## 2. Preliminaries

### 2.1. Problem Definition

In this paper, we focus on simple graph, namely, an undirected graph without self-loops or multiple edges. A labeled graph can be represented as a quadruple *g* = 〈*V*, *E*, *l*_*V*_, *l*_*E*_〉, where *V* is a set of vertices, and *E*⊆*V* × *V* is a set of edges. *l*_*V*_ and *l*_*E*_ are label functions that assign labels to vertices and edges, respectively. *V*(*r*) denotes the vertex set of *r*, and *E*(*r*) denotes its edge set. |*V*(*r*)| and |*E*(*r*)| represent the numbers of vertices and edges in *r*, respectively. *l*_*V*_(*u*) denotes the label of *u* ∈ *V*, and *l*_*E*_(*e*(*u*, *v*)) denotes the label of the edge between *u* and *v*, *u*, *v* ∈ *V*. Additionally, we use |*g* | = |*V* | +|*E*| to depict the size of graph *g*.

**Definition 1 (graph pair).**

A graph pair is a tuple denoted by 〈*r*, *s*〉, where *r* ∈ *R*, *s* ∈ *S*, *R* and *S* are two sets of graph objects, respectively.

**Definition 2 (graph isomorphism).**

A graph *r* is isomorphic to another graph *s*, denoted by *r* = *s*, if there exists a bijection *f* : *V*(*r*) → *V*(*s*) such that

1. ∀*u* ∈ *V*(*r*)(*f*(*u*) ∈ *V*(*s*)∧*l*_*V*_(*u*) = *l*_*V*_(*f*(*u*)));
2. ∀*e*(*u*, *v*) ∈ *E*(*r*)(*e*(*f*(*u*), *f*(*v*)) ∈ *E*(*s*)∧*l*_*E*_(*e*(*u*, *v*)) = *l*_*E*_(*e*(*f*(*u*), *f*(*v*)))).

**Definition 3 (graph edit operation).**

A graph edit operation is an edit operation to transform one graph to another. It can be one of the following six operations:

(i)  insert an isolated vertex into the graph;
(ii)  delete an isolated vertex from the graph;
(iii)  change the label of a vertex;
(iv)  insert an edge between two disconnected vertices;
(v)  delete an edge from the graph;
(vi)  change the label of an edge.

**Definition 4 (graph edit distance).**

The graph edit distance (GED) between graphs *r* and *s*, denoted by GED(*r*, *s*), is the minimum number of edit operations that transform *r* to a graph isomorphic to *s*.

**Example 5 .**

**Figure 1:**
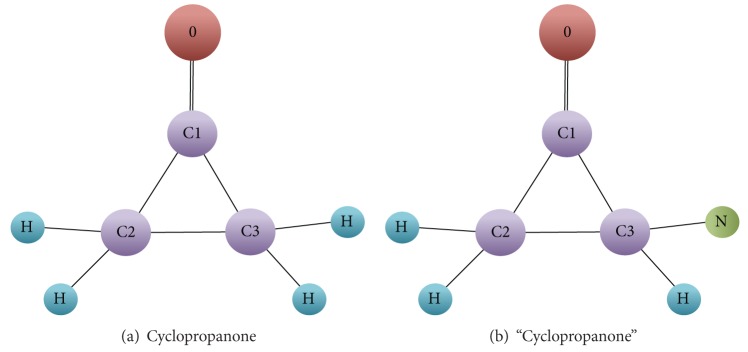
Molecules.

Figure 1(a) illustrates the molecule named cyclopropanone, while Figure 1(b) shows another molecule which does not exist. When recording cyclopropanone into database, errors may be made and the molecule can become the one shown in Figure 1(b). Manual checking is required to find the errors, which is very difficult. Seeing that the two molecules are very similar, we can adapt the GED to measure the similarity so that graph similarity join can be applied to resolve the problem. Take the two molecules as an example. First change the bond (*C*1, *O*) from double to single and then transform one of the atoms *H* bonded with *C*3 into *N*, through which a molecule isomorphic to the one in Figure 1(b) is obtained. So at least two edit operations are required, namely, the graph edit distance. Given the threshold *τ* = 4, the two graphs are regarded similar.

**Problem 6 (graph similarity join).**

Given two sets of graph objects *R* and *S* and a distance threshold *τ* as input, a graph similarity join returns a result set {〈*r*, *s*〉∣*r* ∈ *R*∧*s* ∈ *S*∧GED(*r*, *s*) ≤ *τ*}.

### 2.2. Count Filtering

**Definition 7 (path-based *q*-gram [9]).**

A path-based *q*-gram in a graph is a simple path of length *q*. “Simple” means that there is no repeated vertex in the path.

The path-based *q*-grams of a graph constitute the graph signatures, denoted by *sig* = 〈*V*, *E*, *l*_*V*_, *l*_*E*_〉, where *sig*⊆*r*,|*V* | = *q* + 1, and |*E* | = *q*. Let *Q*_*r*_ be graph *r*'s signature set. We say *sig* and *sig*′ are* common* if *sig* = *sig*′. Note there can be multiple *q*-grams that correspond to one particular signature.

**Lemma 8 (count filtering [9]).**

Graphs *r* and *s* satisfy the distance constraints *τ* if the number of common signatures for 〈*r*, *s*〉 is no less than *LB*:

(1) $$\begin{array}{c} LB=\max\left(\left|Q_{r}\right|-\tau \cdot D\left(r\right),\left|Q_{s}\right|-\tau \cdot D\left(s\right)\right), \end{array}$$

where *D*(*r*) (resp., *D*(*s*)) is the maximum number of affected signatures in *Q*_*r*_ (resp., *Q*_*s*_) when one edit operation is invoked on *r* (resp., *s*).

The count filtering condition check requires *O*(max⁡(*d*_*r*_^*q*^ | *V*(*r*)|, *d*_*s*_^*q*^ | *V*(*s*)|)), where *d*_*r*_ (resp., *d*_*s*_) is the average degree of *r* (resp., *s*). A graph similarity join requires pairwise count filtering condition checks, thus resulting in a complexity of *O*(max⁡(*d*_*r*_^*q*^ | *V*(*r*)|, *d*_*s*_^*q*^ | *V*(*s*)|)|*R*||*S*|). This can be intolerable on large-scale datasets. Next, we present a scalable solution leveraging the MapReduce paradigm.

## 3. Framework

This section presents a MapReduce-based graph similarity join algorithm, following the filtering-verification fashion. The outline of the algorithm is listed below.

### 3.1. Filtering

We allocate two MapReduce jobs to the filtering phase.

*Job 1.* Job 1 counts the same type of common signatures for graph pairs. We use graphs as values and their corresponding *id*s as keys to compose input key-value pairs. *q*-gram signatures (denoted by *sig*) are generated in the Map task. We use the generated signatures as keys and *id*s of graphs as values to form the output key-value pairs. As a consequence, in the Reduce task, we can obtain graphs with common signatures. For the same signature, a graph *id* may appear more than once, since there may exist several *q*-grams in a graph corresponding to an identical signature. The function for Map task is shown as follows.

 Map:〈*r* *id*, *r*〉/〈*s* *id*, *s*〉→〈*sig*, *r* *id*/*s* *id*〉
  1. input 〈*r* *id*, *r*〉 or 〈*s* *id*, *s*〉;
  2. generate *q*-gram signatures for each input graph;
  3. emit 〈*sig*, *r* *id*〉 or 〈*sig*, *s* *id*〉 for each signature with the *id* of its corresponding graph.

The Reducer gets 〈*sig*, *list*(*r* *id*/*s* *id*)〉 in the Reduce task. We define *f*_*r*_ (resp., *f*_*s*_) to denote the occurrences of *r* *id* (resp., *s* *id*) in the *list*(*value*) of Reduce input and *m*_*f*_ to denote the occurrences of graph pairs that share the input key (*sig*) of Reduce, *m*_*f*_ = min⁡(*f*_*r*_, *f*_*s*_). *m*_*f*_ is calculated and output with the corresponding graph pair. The function of Reduce task is as follows.

 Reduce:〈*sig*, *list*(*r* *id*/*s* *id*)〉→〈*r* *id*, (*s* *id*, *m*_*f*_)〉
  1. get list of values consisting of *r* *id* and *s* *id* with the specific key (*sig*);
  2. split the list of values into list of *r* *id* and list of *s* *id*;
  3. for each pair 〈*r* *id*, *s* *id*〉, where *r* *id* is from *list*(*r* *id*) and *s* *id* is from *list*(*s* *id*), calculate *m*_*f*_ and output 〈*r* *id*, (*s* *id*, *m*_*f*_)〉.

*Job 2.* Job 2 counts the total common signatures for graph pairs and checks the count filtering condition, after which the set of candidate pairs is obtained. The Map function is listed as follows.

 Map:〈*r* *id*, (*s* *id*, *m*_*f*_)〉→〈*r* *id*, (*s* *id*, *m*_*f*_)〉
  1. read a 〈*r* *id*, (*s* *id*, *m*_*f*_)〉 into Map task;
  2. emit 〈*r* *id*, (*s* *id*, *m*_*f*_)〉, which is exactly the same as it reads.

We input 〈*r* *id*, (*s* *id*, *m*_*f*_)〉 to the Map task and then output exactly the same key-value pair. Therefore, the Reduce task receives all the graph pairs generated in job 1 with the specific *s* *id*. As the graph pair may have more than one type of common signatures, *m*_*f*_ is summed with the same graph pair for each identical signature, respectively. Subsequently, the graph pairs with less than *LB* (1) common signatures will be discarded. The remaining graph pairs are candidate pairs for verification. The function for Reduce is as follows.

 Reduce:〈*r* *id*, *list*(*s* *id* + *m*_*f*_)〉→〈*r* *id*, *s* *id*〉
  1. receive output from Mappers, the specific *r* *id*, and a list of *s* *id* with *m*_*f*_;
  2. sum *m*_*f*_ and calculate the number of common signatures for each pair 〈*r* *id*, *s* *id*〉;
  3. conduct the count filtering for each pair and output pairs to DFS whose common signatures are more than *LB*.

### 3.2. Verification

In the verification phase, candidate pairs 〈*r* *id*, *s* *id*〉 are to be verified, where the graphs *r* and *s* are required; that is, join operations are necessary to retrieve graphs *r* and *s* by their *id*'s. Hence, we allocate two MapReduce jobs to join (*r* *id*, *r*), (*r* *id*, *s* *id*), and (*s* *id*, *s*).

*Job 1.* Job 1 replaces *s* *id* with graph *s*. The map function takes graph set *S* and 〈*r* *id*, *s* *id*〉 as input and emits 〈*r* *id*, *s*〉, which is listed as follows.

 Map:〈*r* *id*, *s* *id*〉/〈*s* *id*, *s*〉→〈*s* *id*, *r* *id*/*s*〉
  1. input candidate pair 〈*r* *id*, *s* *id*〉 and graph set *S*;
  2. for 〈*r* *id*, *s* *id*〉, emit 〈*s* *id*, *r* *id*〉 and, for 〈*s* *id*, *s*〉, emit it exactly, both of which take *s* *id* as the key.

The Reduce task gathers the list *r* *id* and graph *s* for the key *s* *id* and then outputs the key-value pair 〈*r* *id*, *s*〉. The function for Reducer is as follows.

 Reduce:〈*s* *id*, *list*(*r* *id*/*s*)〉→〈*r* *id*, *s*〉
  1. receive a list of *r* *id* and graph set *S* with the specific key *s* *id*;
  2. for 〈*r* *id*, *s* *id*〉, replace *s* *id* with *s* and output pair 〈*r* *id*, *s*〉.

*Job 2.* Job 2 replaces *r* *id* with graph *r*, invokes label filtering conditions, and calculates GED to find the similar graph pairs.

The function for Map task is as follows.

 Map:〈*r* *id*, *s*〉/〈*r* *id*, *r*〉→〈*r* *id*, *r*/*s*〉
  1. input the candidate pair 〈*r* *id*, *s*〉 and graph set *R*;
  2. emit the key-value pair it reads, where the group key is *r* *id* for both cases.

The function for Reduce task is as follows.

 Reduce:〈*r* *id*, *list*(*r*/*s*)〉→〈*r* *id*, *s* *id*〉
  1. receive a list of values that consisted of graphs *r* and *s* corresponding to the key *r* *id*;
  2. replace *r* *id* with graph *r*;
  3. calculate GED for pairs 〈*r*, *s*〉 and output similar pairs.

### 3.3. Correctness and Complexity Analysis

All graph pairs that satisfy the edit distance constraints are returned error-free, which justifies its correctness.

For algorithm complexity, we take all three phases—Map, Reduce, and Shuffle—into consideration. I/O reading overhead from distributed file system (DFS) is considered for Map task, whereas I/O writing overhead into DFS is analyzed for Reduce task. Both tasks also take time complexity into consideration. Shuffle considers the network communication cost.

Some parameters are defined preceding the analysis. $\bar{\left|g\right|}$ denotes the average size of a graph. *α* means candidate ratio, that is, the percentage of candidate pairs from all graph pairs, and *β* means the ratio of similar pairs from pairs that passed count filtering. We assume that the size of key-value pair that contains only graph IDs is 1.

In job 1 of filter phase, Map reads the data of graph sets *R* and *S* from DFS, the cost of which is $O(\bar{\left|g\right|}\cdot (\left|R\right|+\left|S\right|))$. Consider the worst case in Map task, where *q*-grams are generated for each graph *r*(*s*), and the number of signatures generated is ${\bar{\left|V\right|}}^{q}$, so the time complexity for generating *q*-grams can be estimated as $O({\bar{\left|V\right|}}^{q})$. Therefore, the time complexity for Map task is $O({\bar{\left|V\right|}}^{q}\cdot (\left|R\right|+\left|S\right|))$. As each generated *q*-gram signature forms an output key-value pair (the size for the pair is 1), the communication cost for Shuffle is $O({\bar{\left|V\right|}}^{q}\cdot (\left|R\right|+\left|S\right|))$. In the Reduce task, all graphs represented by *id* containing the same signature are acquired and paired. Thus, the I/O cost is *O*(|*R*||*S*|). Then, the occurrences of *r* *id* and *s* *id* are counted. In other words, all the key-value pairs generated from the Map task are counted, so the time complexity for Reduce is $O({\bar{\left|V\right|}}^{q}\cdot (\left|R\right|+\left|S\right|))$.

Consider job 2 in filter phase, where all the output key-value pairs from job 1 are read into Map task of job 2, so the IO cost is *O*(|*R*||*S*|). Map outputs the key-value pairs it reads, so the time complexity for Map task and communication cost for Shuffle are *O*(|*R*||*S*|). In Reduce task, all pairs are traversed, so the time complexity is *O*(|*R*||*S*|). As we have *α*|*R*||*S*| candidate key-value pairs, the IO cost for Reduce task is *O*(*α*|*R*||*S*|).

In verification phase of job 1, Map reads the candidate pairs represented by 〈*r* *id*, *s* *id*〉 and graph set *S* from DFS, where the IO overhead requires $O(\alpha \left|R\right|\left|S\right|+\bar{\left|g\right|}\left|S\right|)$. The time complexity of Map task and communication cost of Shuffle are also $O(\alpha \left|R\right|\left|S\right|+\bar{\left|g\right|}\left|S\right|)$, because in Map function we just emit what has been exactly input into. Then, in Reduce task, we replace *s* *id* with graph *s*, the time complexity is *O*(*α*|*R*||*S*|), and the IO overhead is $O(\alpha \bar{\left|g\right|}\left|R\right|\left|S\right|)$.

In verification phase of job 2, Map reads the candidate pairs represented by 〈*r* *id*, *s*〉 and graph set *R* from DFS, where the IO overhead requires $O(\bar{\left|g\right|}\left|R\right|(\alpha \left|S\right|+1))$. The time complexity of Map task and communication cost of Shuffle are also $O(\bar{\left|g\right|}\left|R\right|(\alpha \left|S\right|+1))$ for the same reason above. Then, in Reduce task, we first replace *r* *id* with graph *r* and then calculate GED for graph pairs. The GED calculation by the A*-based algorithm requires $O({\bar{\left|V\right|}}^{\bar{\left|V\right|}})$. In the worst case, the time complexity of Reduce is $O(\alpha \left|R\right|\left|S\right|{\bar{\left|V\right|}}^{\bar{\left|V\right|}})$. Finally, similar graph pairs are emitted into DFS, which requires *O*(*αβ*|*R*||*S*|).

## 4. Incorporating Bloom Filters

In the filtering phase of Algorithm 1, two MapReduce jobs are required, with many intermediate key-value pairs generated and transmitted. These increase the I/O and communication cost, which can be fairly time-consuming. This section introduces the Bloom filter technique to reduce such cost. Next, we first recall the concept of spectral Bloom filters.

### 4.1. Spectral Bloom Filter

Bloom filters [12] are space efficient data structures which allow fast membership queries over a given set. A Bloom filter uses *k* hash functions *h*_1_, *h*_2_,…, *h*_*k*_ to hash elements into an array of size *m*. For an element *e* in the set, the bit at positions *h*_1_(*e*), *h*_2_(*e*),…, *h*_*k*_(*e*) in the array is set to 1. Given a query item *q*, we check its membership in the set by examining the bits at positions *h*_1_(*q*), *h*_2_(*q*),…, *h*_*k*_(*q*) of the array. The item *q* is reported to be contained in the set if (and only if) all the aforementioned bits are 1. This method brings a small probability of false-positive; that is, it may return a positive result for an item which actually is not contained in the set but no false-negative while gaining substantial space savings [13].

Spectral Bloom filter (SBF) [14] generalized the basic Bloom filter to be able to record the element frequency, which is thus adopted in this paper. SBF is represented by 〈*A*, *f*〉, where *A* is a set and *f* is a map from *A* to natural numbers; that is, *f* : *A* → *N*, where *N* is the universe of natural numbers. SBF replaces the bit vector with a vector of *m* counters. For insertion of item *e*, the counters *C* = {*C*_*h*_1_(*e*)_, *C*_*h*_2_(*e*)_,…, *C*_*h*_*k*_(*e*)_} are increased by 1 for insertion and decreased by 1 for deletion. Let *f*(*q*) denote the frequency of *q*. A basic query for SBF on an item *q* returns an estimation on *f*(*q*); that is, $\bar{f(q)}=\min_{i\in \{1,\ldots ,k\}}\{C_{h_{i}(q)}\}$. Note that, similar to Bloom filters, SBF also never underestimate *f*(*q*).

### 4.2. Algorithm

Incorporating SBF not only reduces the number of key-value pairs but also contracts the two MapReduce jobs into one in the filtering phase. In particular, the Map task takes graph sets *R* and *S* as input. Then, we create a SBF for each graph by adding the *q*-gram signatures. Cartesian product is conducted for the output key-value pairs 〈*r* *id*, *sbf*_*r*_〉 and 〈*s* *id*, *sbf*_*s*_〉. In the Reduce task, for each graph pair, their SBFs are intersected to estimate the number of common signatures. By intersecting two SBFs with counters *C* ^(1)^ and *C* ^(2)^, respectively, it returns another SBF with counters C*, *C*_i_* = min⁡{*C*_*i*_^(1)^, *C*_*i*_^(2)^}, *i* ∈ {1,…, *m*}. Hence, the number of common signatures could be estimated by ⌊(1/*k*)∑_*i*=1_^*m*^ *C*_i_*⌋. Subsequently, the graph pairs, which have less than *LB* common signatures given by count filtering condition, will be discarded, and the remaining pairs form the candidate set.

We provide the pseudocode of the aforementioned process in Algorithm 2.

### 4.3. Correctness and Complexity Analysis

There is small probability that the false positive case happens with SBF. Specifically, a query for an item *q* in SBF on *f*(*q*), $\bar{f(q)}$ may be larger than *f*(*q*). Therefore, the number of common signatures estimated this way may be larger than the actual value. Nonetheless, false-negative will never happen, which ensures the correctness of the algorithm; in a certain case, the pruning power of count filtering will be impaired. Besides, false-positive will be less likely to happen if one carefully chooses the hash functions and configures the sizes of counters.

Then, we analyze the complexity. Let *m* be the size of a SBF. The Map task reads the entire sets *R* and *S*, so its I/O cost is $O(\bar{\left|g\right|}(\left|R\right|+\left|S\right|))$. Then, signatures are generated and added to SBF, and *k* hash values are calculated for each signature. Thus, the time complexity for Map is $O(k{\bar{\left|V\right|}}^{q}(\left|R\right|+\left|S\right|))$. Map emits the SBF for each input graph, and then Cartesian product is conducted. The communication cost for Shuffle is *O*(*m* ^2^|*R*||*S*|). In the Reduce task, for each graph pair, the number of common signatures is calculated and count filtering condition is checked. Thus, the time complexity is *O*(|*R*||*S*|). Regardless of false-positive cases, the I/O cost is the same as before. Denoting the improved algorithm by “+SBF,” we summarize the complexity results in Table 1.

## 5. Optimizing Verification Phase

In verification phase, we need to calculate the GED of candidate pairs. Nevertheless, it is not capable of finishing the calculation with large graphs and threshold *τ*. Therefore, we devise a MapReduce-based method for GED calculation, which is able of handling large-scale graphs. Besides, join operations are required preceding the GED calculation to get the entire graph. Thus, three relations *R*(*r* *id*, *r*)⋈*C*(*r* *id*, *s* *id*)⋈*S*(*s* *id*, *s*) are joined to obtain the input of the GED algorithm, where *C*(*r* *id*, *s* *id*) is the output of the filtering phase. Inspired by the idea of multiway join, we can reduce the number of required MapReduce jobs from two to one.

### 5.1. MapReduce for GED Calculation

The GED calculation is based on A* algorithm. A* constructs a search-tree, the node of which represents a mapping status. A mapping status is stored in an array (denoted by *x*), the index of which stands for different vertices in graph *r* and the corresponding value stands for the vertices of graph *s*. A* explores the space of all possible vertex mappings between two graphs in a best-first search fashion with function (denoted by *f*(*x*)) established to determine the order in which the search visits vertex mappings. *f*(*x*) is a sum of two functions: (1) the distance from the initial state to the current state (denoted by *g*(*x*)); (2) a heuristic estimate of the distance from the current state to the goal (denoted by *h*(*x*)). *g*(*x*) and *h*(*x*) are calculated by the following equations:

(2) g(x)=GED(rp,sp);h(x)=Γ(LV(rp)LV(sq))+Γ(LE(rq),LE(sq)).

*r*_*p*_ consists of the vertices that have been mapped and edges connecting them, while *r*_*q*_ consists of the vertices unmapped yet as well as their resident edges. The equation for *h*(*x*) represents the label difference of two graphs.

The search space for A*-based approach is very large, requiring $O({\bar{\left|V\right|}}^{\bar{\left|V\right|}})$. In order to boost up the searching procedure, parallelization is a common way to think about. The naive way is to allocate different branches of search-tree to different workers so that the searching procedure can proceed in parallel. However the load is not balanced this way so that the final runtime is determined on the worker with the heaviest load. As a consequence, we devise a MapReduce-based method to calculate GED, denoted by MRGED, which reallocates the works after each MapReduce round.

The format of the key-value pairs to be manipulated by MapReduce is 〈*x*, *f*(*x*)〉. The procedure of searching for the result is through iterations that each iteration walks down one layer of the search-tree.

### 5.2. Multiway Join

In relational database, a multiway join can process *T*_1_(*A*, *B*)⋈*T*_2_(*B*, *C*)⋈*T*_3_(*C*, *D*) together in one round, where *T*_*i*_(*X*, *Y*) is a relational table with attributes *X* and *Y* (Algorithm 3). Following the same idea, we can consolidate the two MapReduce jobs required for verification in Algorithm 1. Specifically, let *h* be a hash function with range 1,2,…, *n*, where *n* ^2^ is the number of Reducers. We associate each Reduce task with a pair (*i*, *j*), where *i*, *j* ∈ [1, *n*]. Each tuple *t*_2_(*b*, *c*) ∈ *T*_2_(*B*, *C*) is sent to the Reducer numbered (*h*(*b*), *h*(*c*)), while each tuple in *T*_1_(*a*, *b*) (resp., *T*_3_(*c*, *d*)) is sent to Reducers numbered (*h*(*b*), *x*) (resp., (*y*, *h*(*c*))), for any *x* (resp., *y*). Each Reduce task computes the join of the tuples it receives. It is shown that multiway join is more efficient in practice than two simple joins [15].

We encapsulate the improved verification procedure in Algorithm 4.

### 5.3. Correctness and Complexity Analysis

One may immediately verify that Algorithm 4 correctly conducts the verification.

In the multiway join based verification phase, the Map task takes graph sets *R* and *S* and the candidate pairs represented by their *id*s as input. The input I/O cost is $O(\bar{\left|g\right|}(\left|R\right|+|S|)+\alpha \left|R\right|\left|S\right|)$. The key-value pair 〈*r* *id*, *r*〉 (resp., 〈*s* *id*, *s*〉) is sent to *n* Reduce tasks numbered (*r* *id*, *x*) (resp., (*y*, *s* *id*)) for any *x* (resp., *y*), whereas the key-value pair 〈*r* *id*, *s* *id*〉 is sent to the only Reduce task numbered (*h*(*r* *id*), *h*(*s* *id*)). As a result, the communication cost for Shuffle is $O(n\bar{\left|g\right|}(\left|R\right|+\left|S\right|)+\alpha \left|R\right|\left|S\right|)$, where *n* ^2^ is the number of Reducers. In the Reduce task, all candidate graph pairs go through edit distance computation. Pairs with larger size go through MRGED, while pairs with smaller size go through A*. For simplicity, the complexity of GED calculation is regarded the same as the baseline algorithm. Labelling the resulting algorithm with “+MJ,” we summarize the complexity results in Table 2.

## 6. Experiments

### 6.1. Experiment Setup

We conducted experiments on several publicly available real datasets but only present the results on Pubchem (http://pubchem.ncbi.nlm.nih.gov) due to the interest of space. The dataset is constructed by sampling 1,000,000 graphs from Pubchem.

Amazon cloud services were used as our experiment platform. Specifically, we used Elastic Compute Cloud (EC2), in which the computing nodes are called instances. In the experiment 31 instances were used by default—one set as master node and others as worker nodes. The standard configuration of all EC2 instances is m1.small, one CPU of single core with 1.7 GB memory running Hadoop 1.1.2 (Table 3).

### 6.2. Evaluating Filters

In order to evaluate the effectiveness of our filtering techniques, we use the term “Basic” for the baseline algorithm for processing graph similarity joins based on MapReduce. “+SBF” denotes filtering improved algorithm of Basic by incorporating SBF.

**Figure 2:**
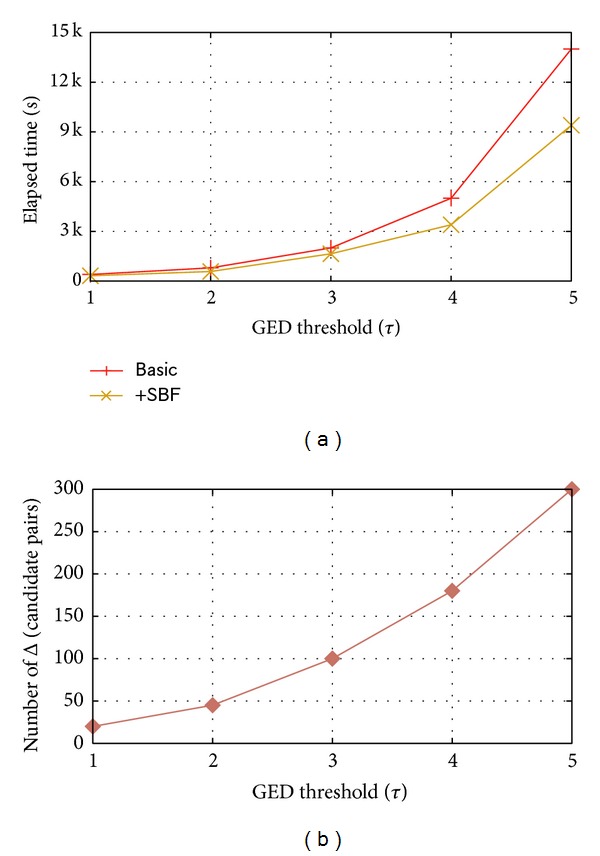
Evaluating filtering.

The algorithm efficiency has been studied and shown in Figure 2(a). However, the pruning power is somewhat impaired, so we conducted the experiment to record the increase of candidate pairs through “+SBF” (cf.  Figure 2(b)). It can be revealed that “+SBF” outweigh “Basic” in efficiency by sacrificing little pruning power. When *τ* = 5, less than 300 more candidate pairs are generated while about 5,000 seconds are reserved.

### 6.3. Evaluating Verification

**Figure 3:**
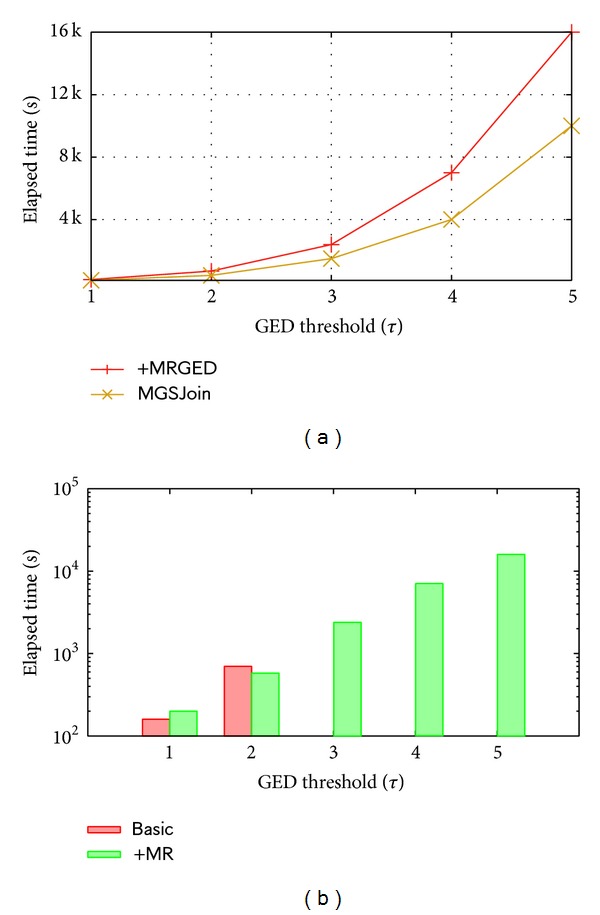
Evaluating verification.

The verification was evaluated with candidate pairs generated by Basic. Term “+MJ” denotes applying multiway join in verification, while “+MRGED” denotes adapting alternative MapReduce-based GED calculation. We use term “MGSJoin” to indicate the basic algorithm incorporating both techniques.  Figure 3(a) shows the runtime comparison between +MRGED and MGSJoin. The result illustrates the superiority of applying multiway join. When *τ* = 5, algorithm with multiway join is about 6,000 seconds faster than with ordinary joins.  Figure 3(b) presents the result of evaluating MRGED, where Basic and +MRGED are compared. It can be observed that the runtime of both algorithms grows exponentially. When *τ* equals 1, the Basic finishes quicker than +MRGED (162 s and 204 s, resp.), while *τ* equals 2; the +MRGED outweighs Basic (the runtime is 712 s and 580 s, resp.). This is because the calculation required for *τ* = 1 is small, where the MRGED is clumsy compared with A*, whereas when *τ* = 2 more calculation is required so that the advantage of MRGED comes out. When *τ* is within the range of 3–5 Basic is unable to finish because the large calculation drives Basic out of memory.

### 6.4. Comparing with State-of-the-Art Method

**Figure 4:**
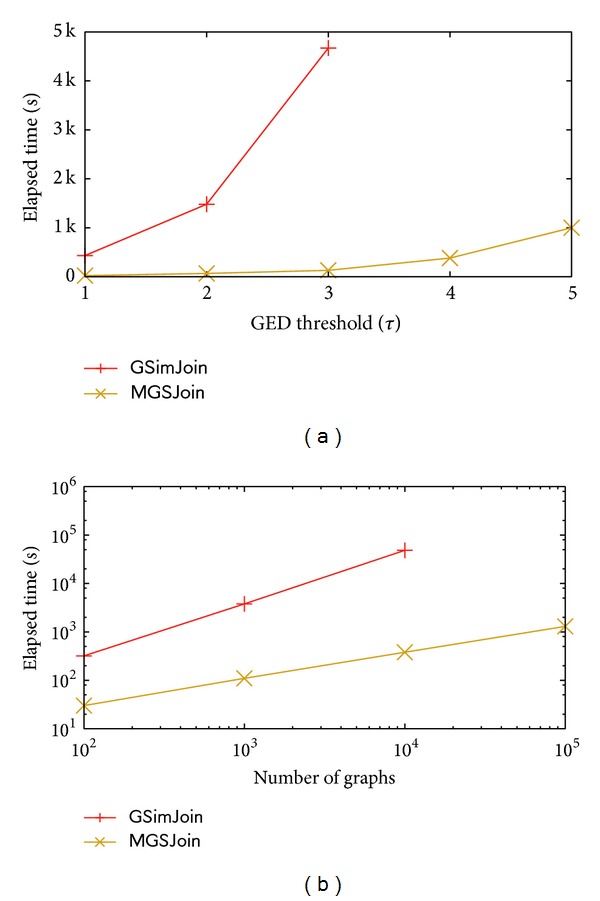
Comparing with state-of-the-art method.

We compared our algorithm with the state-of-the-art method, GSimJoin. In Figure 4(a), we chose 10,000 graphs in order to compare with GSimJoin and the result witnesses the obvious superiority of MGSJoin over GSimJoin.  Figure 4(b) is drawn in log scale, which varies the number of graphs and records the elapsed time. Both algorithms grow linear in the figure, which reflect their exponential growth. MGSJoin rises mush slower than GSimJoin. The runtime of GSimJoin is about 10 times longer than MGSJoin when joining 100 graphs and 100 times longer than MGSJoin when joining 10,000 graphs. Moreover, when we have to join 100,000 graphs, GSimJoin is running out of memory so that no result is recorded.

### 6.5. Speedup

**Figure 5:**
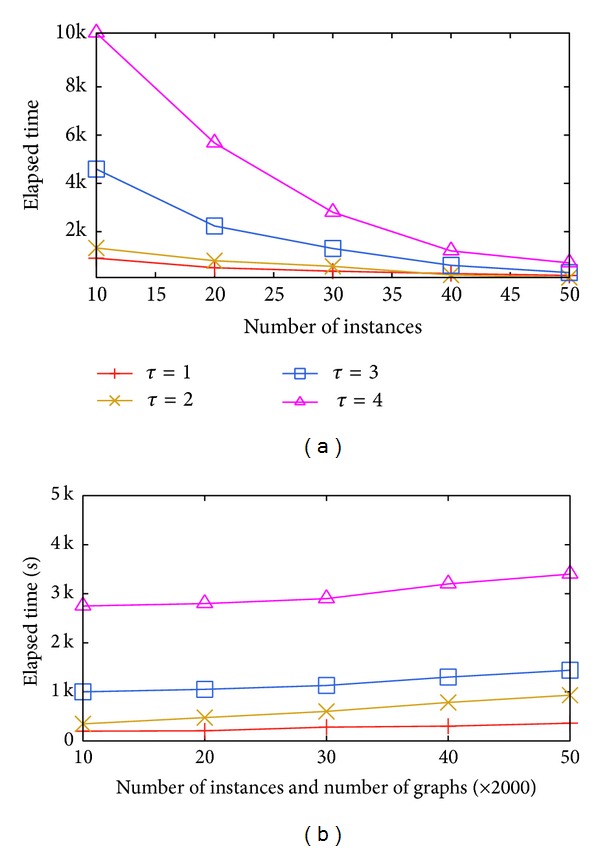
Speedup and Scale-up.

We evaluated the speedup of our algorithm by varying the number of instances from 10 to 50. The experimental results are shown in Figure 5(a). We can see that, with the increase of instances in the cluster, the performance of MGSJoin significantly improved. The improvement is significantly shown when threshold *τ* equals 4. With more instances running, the count filtering is getting faster by counting for the common signatures simultaneously and the verification is getting quicker by joining relations and calculating GED in parallel.

### 6.6. Scale-Up

We evaluated the scale-up of our algorithm by increasing both dataset sizes and number of nodes in the cluster. The result is shown in Figure 5(b). It is worth noting that as the dataset increased the results for different values of *τ* get similar in the trend of increase. All lines rise smoothly, which reveals good scalability of MGSJoin.

## 7. Related Work

*Graph Similarity Queries*. Similarity joins retrieve similar data object pairs, which can be strings, sets, trees, and graphs [16]. As to GED-based graph similarity search, [17] proposed *κ*-AT, a tree-based *q*-gram approach. However, it is associated with the drawback of usually loose lower bound for count filtering. Seeing the drawback, [9] presented a path-based *q*-gram approach. In comparison with *κ*-AT, GSimJoin is more efficient by leveraging more advanced filtering techniques. Thus, we adopt the path-based *q*-gram approach in this paper.

*MapReduce-Based Graph Algorithms*. MapReduce is a distributed programming framework [10], which has been applied in processing large graphs. Many graph algorithms using MapReduce were discussed in [18], including triangles/rectangles enumeration and *k*-cliques computation. In [19], several techniques were proposed to reduce the input size of MapReduce, and the techniques are applied for minimum spanning trees, approximate maximal matchings, approximate node/edge covers, and minimum cuts. Personalized PageRank computation in MapReduce was discussed in [20]. Matrix multiplication based graph mining algorithms in MapReduce were investigated in [21]. More recently, densest subgraph computation [22], subgraph instances enumeration [23], and connected components computation in logarithmic rounds [24] were researched in MapReduce. 

*Graph Processing Systems in Cloud*. Many systems were developed in order to deal with big graphs. Such a one representative system is Pregel [25], which takes a vertex-centric approach and implements a bulk synchronous parallel (BSP) computation model. HipG [26] improves BSP by using asynchronous messages to avoid synchronization. PowerGraph [27] is a distributed graph processing system that is optimized to process power-law graphs. Giraph++ was proposed in [28] to take graph partitioning into consideration when processing graphs. Workload balancing for graph processing in cloud was discussed in [29].

## 8. Conclusion

In this paper, we have investigated the problem of scalable graph similarity joins. We firstly present a MapReduce-based graph similarity join algorithm MGSJoin following the filtering-verification framework. To reduce the communication cost in the filtering phase, it incorporates the Bloom filter technique to reduce the number of intermediate key-value pairs. In addition, we devise a multiway join optimized verification procedure for further speedup. Extensive experiments are conducted on real datasets to confirm the efficiency and scalability of the proposed solution. Furthermore, the verification phase is further optimized with MapReduce, which enables the test on larger and denser graphs.

As a future direction, we plan to explore the possibility of optimizing the verification with multithreaded programming paradigm. Additionally, it is also of interest to test the efficiency and scalability of proposed algorithms on even larger and/or denser graphs.

## Figures and Tables

**Algorithm 1:**
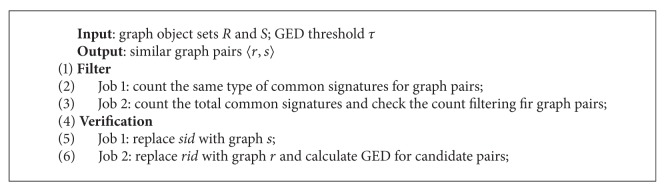
MGSJoin.

**Algorithm 2:**
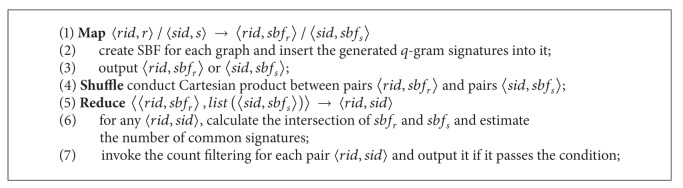
Replacement of filter of Algorithm 1.

**Algorithm 3:**
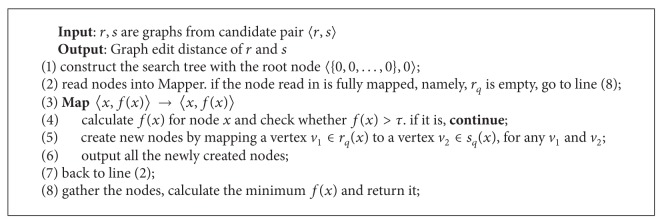
MRGED (*r*, *s*).

**Algorithm 4:**
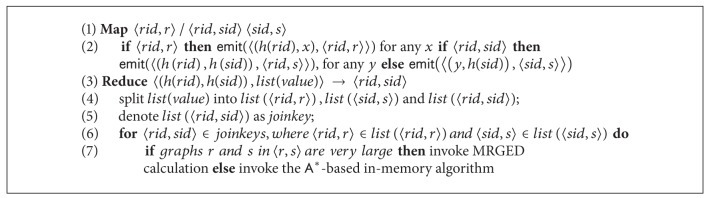
Replacement of verification of Algorithm 1.

**Table 1:** Complexity analysis of filtering.

Phase		Job 1	Job 2	+SBF
Map	I/O	$O\left(\bar{\left|g\right|}\cdot \left(\left|R\right|+\left|S\right|\right)\right)$	*O*(|*R*||*S*|)	$O\left(\bar{\left|g\right|}\cdot \left(\left|R\right|+\left|S\right|\right)\right)$
	Time	$O\left({(\bar{\left|V\right|}}^{q}\cdot (|R|+|S|)\right)$	*O*(|*R*||*S*|)	$O\left({k\bar{\left|V\right|}}^{q}\cdot \left(\left|R\right|+\left|S\right|\right)\right)$

Shuffle		$O\left({(\bar{\left|V\right|}}^{q}\cdot (|R|+|S|)\right)$	*O*(|*R*||*S*|)	*O*(*m*|*R*||*S*|)

Reduce	I/O	*O*(|*R* | |*S*|)	*O*(α|*R*||*S*|)	*O*(α|*R*||*S*|)
	Time	$O\left({(\bar{\left|V\right|}}^{q}\cdot (|R|+|S|)\right)$	*O*(|*R*||*S*|)	*O*(|*R*||*S*|)

**Table 2:** Complexity analysis of verification.

Phase		Job 1	Job 2	+MJ
Map	I/O	$O\left(\alpha \left|R\right|\left|S\right|+\bar{\left|g\right|}\left|S\right|\right)$	$O\left(\bar{\left|g\right|}\left|R\right|\cdot \left(\alpha \left|S\right|+1\right)\right)$	$O\left(\bar{\left|g\right|}\left(\left|R\right|+\left|S\right|\right)+\alpha \left|R\right|\left|S\right|\right)$
	Time	$O\left(\alpha \left|R\right|\left|S\right|+\bar{\left|g\right|}\left|S\right|\right)$	$O\left(\bar{\left|g\right|}\left|R\right|\cdot \left(\alpha \left|S\right|+1\right)\right)$	*O*(*n*(|*R*| + |*S*|) + α|*R*||*S*|)

Shuffle		$O\left(\alpha \left|R\right|\left|S\right|+\bar{\left|g\right|}\left|S\right|\right)$	$O\left(\bar{\left|g\right|}\left|R\right|\cdot \left(\alpha \left|S\right|+1\right)\right)$	$O\left(n\bar{\left|g\right|}\left(\left|R\right|+\left|S\right|\right)+\alpha \left|R\right|\left|S\right|\right)$

Reduce	I/O	$O\left(\alpha \bar{\left|g\right|}\cdot \left|R\right|\left|S\right|\right)$	*O*(αβ|*R*||*S*|)	*O*(αβ|*R*||*S*|)
	Time	*O*(α|*R*||*S*|)	$O\left(\alpha |R||S{\bar{\left|V\right|}}^{\bar{\left|V\right|}}\right)\;$	$O\left(\alpha |R||S{\bar{\left|V\right|}}^{\bar{\left|V\right|}}\right)\;$

**Table 3:** Dataset statistics.

Dataset	|*R*|	|*V*|	|*E*|	Disk size (GB)
Enamine	1,000,000	52.32	50.37	0.7
